# Illness Perceptions in Women with Breast Cancer—a Systematic Literature Review

**DOI:** 10.1007/s12609-015-0187-y

**Published:** 2015-08-07

**Authors:** Ad A. Kaptein, Jan W. Schoones, Maarten J. Fischer, Melissa S. Y. Thong, Judith R. Kroep, Koos J. M. van der Hoeven

**Affiliations:** Medical Psychology, Leiden University Medical Center (LUMC), PO Box 9600, 2300 RC Leiden, The Netherlands; Walaeus Library, LUMC, PO Box 9600, 2300 RC Leiden, The Netherlands; Clinical Oncology, LUMC, PO Box 9600, 2300 RC Leiden, The Netherlands; Medical and Clinical Psychology, Tilburg University, PO Box 90 153, 5000 LE Tilburg, The Netherlands

**Keywords:** Breast cancer, Illness perceptions, Quality of life, Cognitions, Emotions, Systematic literature review

## Abstract

Women with breast cancer respond to the illness and its medical management in their own personal way. Their coping behavior and self-management are determined by their views (cognitions) and feelings (emotions) about symptoms and illness: their illness perceptions. This paper reports the results of a systematic literature review of illness perceptions and breast cancer. In the 12 studies identified, published between 2012 and 2015, illness perceptions were found to be important concomitants of medical and behavioral outcomes: fear of recurrence, distress, quality of life, satisfaction with medical care, use of traditional healers, and risk perception. Intervention studies are called for where the effects are examined of replacing unhelpful illness perceptions by more constructive ones. Health care providers do well by incorporating illness perceptions in their care for women with breast cancer, as this is instrumental in improving patients’ quality of life.

## Introduction

Physicians are well aware of how being ill elicits behavioral, psychological and social reactions that shape the lives of the patients, and of those in his or her social environment. Incorporating these reactions into the medical management of patients is nowadays almost routine. This statement is supported by the use of methods to assess quality of life (QOL) and “patient-reported outcomes” (PRO) [1•, 2]. In modern medicine, QOL and PRO are not merely buzz words, but they lay the foundation for patient centered care, with shared decision making and self-management skills that help improve patients’ QOL [e.g., 3•, 4].

Improving QOL of patients is not a straightforward part of the medical management of patients with breast cancer. Multicolored brochures, fancy video films, elaborate technological fads, or—better—a dedicated conversation in the doctor’s office between doctor and patient is not necessarily effective. Every physician will have experienced how medical explanations of possible causes and treatments, diagnostic and therapeutic procedures, or side effects of medication seem to be falling on deaf ears of quite a few patients. Behavioral medicine offers explanations for this quite often frustrating but also fascinating phenomenon.

Hippocrates was right: “If you miss being understood by laymen, and fail to put your hearers in this condition, you will miss reality” [in 5, p. ii]. Fortunately, modern research can help in preventing our message falling on deaf ears—by putting ourselves in the position of the patient and the story she tells herself and her physician about her breast cancer. Kleinman, MD and anthropologist, sat in cafes in Taiwan and asked customers about their physical complaints. He found out that the stories people told him about their health shared five components: What is it, what causes it, what can I do about it, what can the physician do about it, and how long will it last? Interestingly, people in North America and Europe asked themselves exactly the same questions about their physical problems [5]. Their “explanatory model” or illness narrative had a similar structure see also [6]. Explanatory models may very well be medically incorrect—they nevertheless drive behavior toward symptoms and medical treatment (e.g., attending screening campaigns, adhering to medication). Kleinman suggests eliciting the patient’s explanatory model by asking:

“(1) What do you think has caused your problem? (2) Why do you think it started when it did? (3) What do you think your sickness does to you? How does it work? (4) How severe is your sickness? Will it have a short or a long course? (5) What kind of treatment do you think you should receive? (6) What are the most important results you hope to receive from this treatment? (7) What are the chief problems your sickness has caused for you?, and (8) What do you fear most about your illness?” ([7], p. 256).

Health care providers who dismiss explanatory models as “unscientific” do not adhere to Hippocrates’ dictum—and will most likely be less successful in improving patients’ QOL compared with their colleagues who stick to the advice of one of the founding fathers of medicine [1•].

**Fig. 1:**
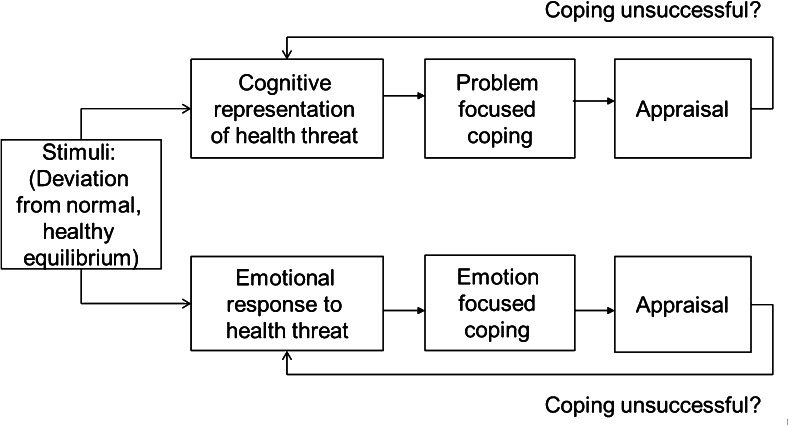
Self-regulation model (SRM) [36]

Modern empirical research in behavioral medicine introduced the concept of “illness perceptions” as the key to studying, understanding, and addressing explanatory models of patients. Illness perceptions are defined as “the cognitive (i.e., beliefs, ideas, thoughts) and emotional (i.e., feelings) representations of symptoms and illnesses” [8]. A woman who believes that breast cancer is caused by stress and emotions will not attend breast cancer screening: “screening will not take away my stress.” A woman who thinks that her breast cancer cannot be treated effectively will stay at home when her chemotherapy session in the hospital is scheduled. A physician who tells these women that they are wrong (worse: stupid) “… misses being understood by laymen, and … will miss reality.” A physician, on the other hand, who explores the illness perceptions of these women and attempts to change the perceptions into more adaptive thoughts (cognitions) and feelings (emotions), is most likely successful in increasing attendance at breast cancer screening and breast cancer treatment. The self-regulation model (SRM) encompasses the elements in an elegant model that we described above clinically (Fig. 1).

Physical sensations, perceived to be deviating from normal, are labeled in a cognitive and an emotional manner. Note how this labeling does *not* equal “correct information”— representations differ from “objective knowledge.” Illness representations are shaped not only by the contacts patients have with health care providers but first and foremost by contacts with laymen, television, women’s magazines, family traditions, and dominant stories in cultures. Whether these cognitions are “correct” or not is irrelevant: “feelings are facts” [9]. Note how this is true for physicians as well: medical views on (breast) cancer of 50 years ago were presented with all honesty and power by physicians at that time. Nowadays, physicians tend to look rather critically at those views. In 50-year time from now, similar responses will most likely be observable regarding current dominant medical views on breast cancer.

Illness perceptions can be assessed via psychometrically sound questionnaires, in particular the Illness Perception Questionnaire—Revised IPQ-R, [10] and the Brief Illness Perception Questionnaire B-IPQ [11]; www.uib.no/ipq contains extensive information on all aspects of illness perception questionnaires.

An innovative way of assessing illness perceptions is via asking patients to draw their illness [12]. A study of patients surviving a myocardial infarction showed how the drawings by patients predicted symptoms of angina, resumption of social activities, and return to work better than laboratory and clinical measures. In cancer, we compared the drawings patients made of their lung cancer with the actual X-thorax which showed the tumors. It was found that patients drew their tumors larger than they actually were; also, the more accurate the drawing was, the higher the sense of pessimism in the patient [9].

A fascinating third method was used in a study by Harrow et al., where women with breast cancer were asked to represent their breast cancer using clay. The women formed the clay according to what they felt their breast cancer was like. It was found that “almost all women had a mental image of their cancer. Images reflected their *beliefs* about their illness (its appearance, character, and dangerousness) and appeared to be related to a number of *fears and concerns*. The origin of images was uncertain but appeared to be influenced by scan images, verbal metaphors presented by health professionals, and previous beliefs held about cancer. Some women used metaphors presented to infer properties of the cancer that may have been unintended by the health professional” [13], emphasis in the original paper.

A fourth also somewhat unconventional method to assess illness perceptions pertains to studying novels, poems, music, films, and paintings on how illnesses are represented in those art genres. We applied this approach in an analysis of the illness perceptions about cancer in *Cancer Ward* by Solzhenitsyn [14]. A study on novels about breast cancer is still waiting to be performed.

Further to the questions that Kleinman et al. suggest, doctors should discuss with their patients, and it is highly instructive to read the content of the eight questions (items) that make up the B-IPQ:

How much does your illness affect your life?

How long do you think your illness will continue?

How much control do you feel you have over your illness?

How much do you think your treatment can help your illness?

How much do you experience symptoms from your illness?

How concerned are you about your illness?

How well do you feel you understand your illness?

How much does your illness affect you emotionally? (e.g., does it make you angry, scared, upset or depressed?)

Clearly, the questions suggested by Kleinman et al. are covered to a great degree by the items in the B-IPQ. Clinicians and researchers have, therefore, quite a few approaches at their disposal to assess patients’ illness perceptions.

The aim of the current paper is to review the research on illness perceptions in women with breast cancer, published since 2012, with a view to presenting an overview of the state-of-the-art in the area, examine the associations between illness perceptions and medical and behavioral outcomes, and discuss the research and clinical implications of our findings. An earlier paper presented a comparable review of the research up to 2012 [15].

## Method

We performed a search in PubMed, MEDLINE (OVID-version), Embase (OVID-version), Web of Science, COCHRANE Library, CINAHL (EbscoHost-version), and PsycINFO (EbscoHost-version). The search consisted of the combination of two subjects:

- Illness representations
- Breast cancer

**Fig. 2:**
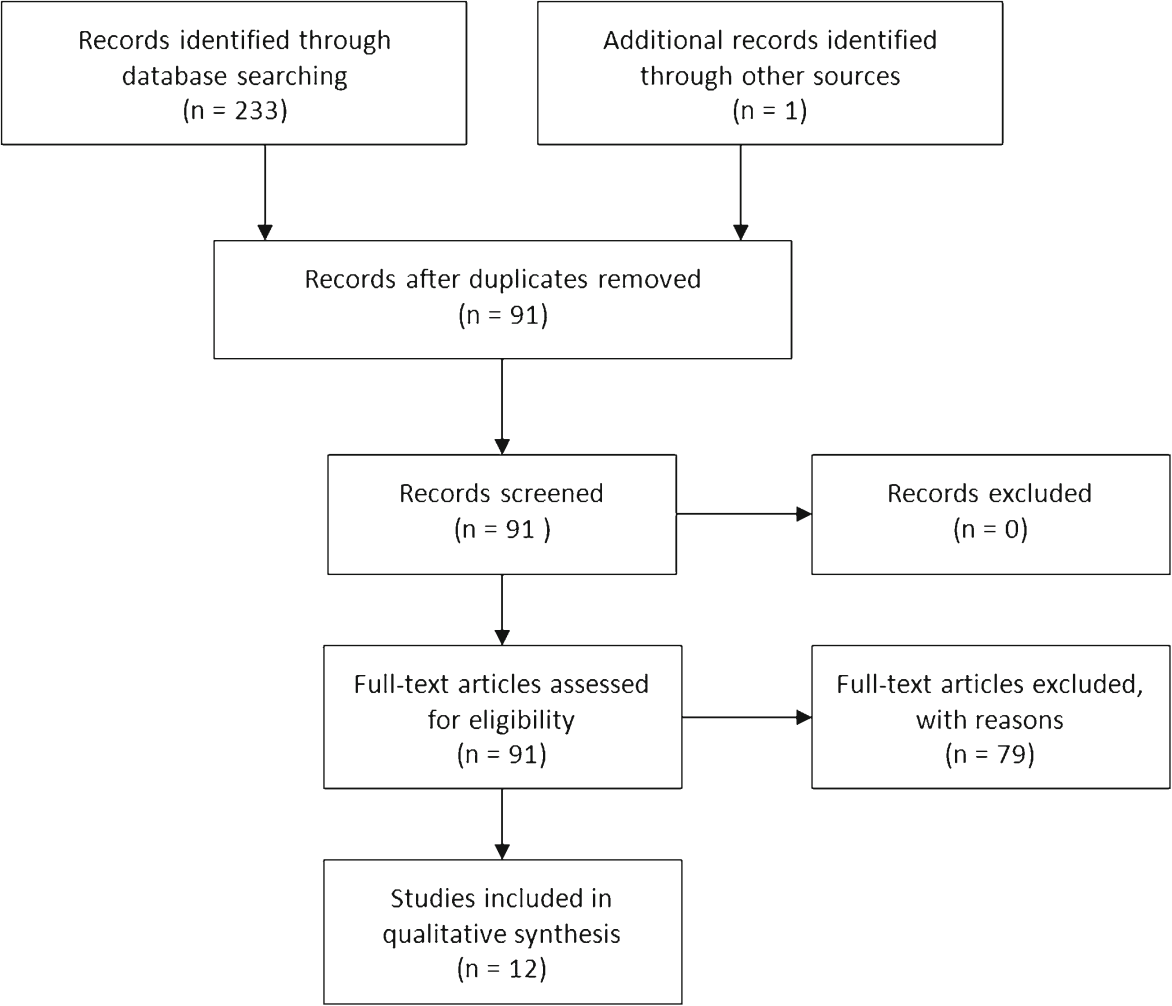
Flow diagram literature search

The query was applied in all databases taking into account the terminological and technical differences between these databases. Various synonyms and related terms for all subjects were used. Detailed search strategies can be found in the Appendix Table 2. The final search was performed on the 12th of March 2015. Results were limited to articles in the English language and from the year 2004 onwards. The databases yielded 90 references in total. We selected papers, written in English, which were published as of January 1, 2012, up to March 12, 2015 (Table 1). This time-window was chosen because an earlier publication from our group reviewed the subject of study until December 31, 2011 [15] and at the request of the Journal. Exclusion criteria were meeting abstract publication, healthy women as respondents, patient groups that included patients with other cancer types than breast cancer, and behavioral aspects of mammography. The flow chart below (Fig. 2) details the search strategy and selection process (see also Appendix Table 2).

**Table 1:** Results of literature review of studies on illness perceptions in women with breast cancer, January 1, 2012, until present

First author, year of publication, country of origin, reference	Number of patients mean age (SD)	Clinical characteristics	Design	Questionnaire used to assess illness perceptions	Results
Charlier 2012 BEL [29]	440 52 (8)	Survivors of primary non-metastatic breast cancer	Cross-sectional	IPQ-R	Illness perceptions are part of clusters that differentiate patients’ responses to their illness. The clusters are associated with levels of physical activity and supportive care needs: distress was associated with participants in the onco-revalidation
Corter 2013 NZ [30]	153 ∼58	Time since diagnosis 3.3 years	Cross-sectional	B-IPQ	Fear of recurrence associated with B-IPQ scores (i.e., low treatment control, more negative emotions, longer timeline, more symptoms), and with medication necessity beliefs
Fischer 2013 NL [3•]	57 50.7 (6.9)	Curative medical treatment	Longitudinal	B-IPQ	Distress at conclusion of aftercare program was predicted by changes in illness perceptions, i.e., stronger chronic and cyclical timeline perceptions, more symptoms attributed to breast cancer
Fischer 2015 NL [26]	19 53.8 (10.0)	Primary breast cancer surgery and/or radiation	Longitudinal	B-IPQ	A Nordic Walking group aftercare exercise intervention reduced arm and shoulder symptom severity, perceived consequences of the symptoms, and perceived symptom severity
Iskandarsyah 2013 IND [18]	70 45 (7.9)	Stage III and IV breast cancer	Cross-sectional	B-IPQ	Illness perceptions are associated with treatment satisfaction and quality of life: High quality medical communication was associated with stronger personal control, lesser concerns, better understanding of illness, and less emotionally affected by the illness
Iskandarsyah 2014 IND [19]	70 45.6 (7.9)	50 % stage III and IV	Cross-sectional	B-IPQ	Missing treatment sessions associated with more negative illness perceptions. Consulting a traditional healer and more negative illness perceptions associated with missing treatment sessions. B-IPQ total: more negative means more distress
Kaptein 2013 NL, JP [15]	43 D 47 (7.8) JP 50 (9.6)	Ductal carcinoma	Longitudinal	B-IPQ	Japanese women scored higher on “concern” and “coherence” than Dutch women. On other illness perception dimensions difference were minor. Breast cancer illness perceptions more negative and threatening than in patients with asthma, diabetes
McCorry 2013 IRL [31]	90 57.2 (10.4)	Tumor grade 2 and 3	Longitudinal	IPQ-R	Illness perceptions remained relatively stable 6 months post-diagnosis. Illness perceptions predicted distress. Patients with negative illness perceptions and poor coping reported high distress.
Petrie 2015 NZ [32•]	2269 55.5 (9.1)	Early stage breast cancer	Cross-sectional	B-IPQ	Decision regarding bilateral mastectomy was associated with genetic or hormonal causal beliefs about breast cancer.
Ploos van Amstel 2013 NL [33]	129 57 (10)	Curative treatment for breast cancer	Longitudinal	ICQ	Illness cognitions are associated with distress. Reduced quality of life, reduced cognitive function (helplessness, denial), and fatigue predicted distress
Silva 2012 POR [34]	78 52.1 (8.9)	Radiotherapy 27 %, Chemotherapy 73 %	Cross-sectional	B-IPQ	Posttraumatic growth buffers treatment-related distress and consequences. High posttraumatic growth associated with lower depression and higher quality of life
Thomson 2014 AUS [35]	1109 (∼60)	No details reported	Cross-sectional	RPQ	Causal beliefs about breast cancer are related to stress and life style

*BEL* Belgium, *NZ* New Zealand, *NL* the Netherlands, *IND* Indonesia, *JP* Japan, *IRL* Ireland, *POR* Portugal, *AUS* Australia, *B*-*IPQ* brief illness perception questionnaire, *ICQ* illness cognition questionnaire, *IPQ*-*R* illness perception questionnaire revised, *RPQ* risk perception questionnaire

## Results

The 12 studies that resulted from the literature search are depicted in Table 1. The Netherlands is the country where most studies in this sample originate from. This is not really surprising as breast cancer in that country has one of the highest prevalence rates in the world. In addition, patients, health care providers, and patient organizations have a long tradition of including psychosocial issues in the medical management of breast cancer. The number of patients in the studies selected is quite substantial, ranging from 43 to 2269. IPQ-R and B-IPQ are the questionnaires that are used most often to assess the illness perceptions.

In an earlier paper, we reviewed illness perceptions in women with breast cancer as well, from the first study in 1996 to 2013, with in essence similar results [15]. Therefore, that paper and the current one represent a summary of illness perception research in women with breast cancer. We identified 12 studies in the current paper, spanning a three and a half year period; its predecessor identified 14 studies, over a period spanning 16 years (1996 to 2011). It seems, therefore, that 26 papers represent the empirical research on illness perceptions in women with breast cancer, up until mid-2015.

The results of the selected studies are fairly straightforward: Illness perceptions turn out to be clearly associated with major outcomes, i.e., symptoms, fear of recurrence, distress, QOL, satisfaction, adherence to treatment, seeking help from traditional healers, coping, mastectomy, and risk perception.

A number of observations regarding this overall result are in place. In the identified studies, the dependent variable or variables range from levels of physical activity, supportive care needs, fear of recurrence, distress, quality of life, adherence to chemotherapy, and decisions about bilateral mastectomy. These outcomes are of major importance in the lives of the patients and reflect patient-reported outcomes (PRO, rather than outcomes such as tumor volume or length of survival). The studies that we review here indicate that in the cross-sectional studies, the PRO measures are associated with various dimensions of illness perceptions. In the longitudinal studies, results even point out how illness perceptions appear to predict (or influence) the various dependent variables (PRO). Given the relatively small number of studies, it would be overstating the case if we would conclude that illness perceptions cause changes in various PRO measures. At the same time, in our previous paper on this topic and in research on illness perceptions in other chronic somatic disorders, the intervention by Petrie et al. [16] was used to illustrate how addressing maladaptive illness perceptions resulted in positive changes in major outcomes such as symptoms, return to work, and resumption of sexual activity. In patients with breast cancer, the research group of Antoni et al. publishes studies that appear to illustrate the benefits of cognitive-behavioral therapy [1•]. Recent work by Aaronson et al. corroborates these findings [17].

In our literature review, it is not always clear to what degree which illness perception dimension is associated with which outcome measure. Statements about these associations require very large patient samples and probably more experimental designs.

## Discussion and Conclusion

Two major results stand out from this review. Illness perceptions research in women with breast cancer is a topic with increasing attention and relevance in behavioral medicine research. Secondly, illness perceptions in women with breast cancer are associated with major outcomes in the course of the illness. As in comparable illness perception research, clinical and sociodemographic characteristics are hardly, if at all, associated with illness perceptions. This is consistent with the self-regulation model, where it is explained how illness perceptions are shaped and influenced by how people perceive and make sense of the world around them. Illness perceptions guide people/patients in their health behavior and illness behavior. Illness perceptions vary per individual and per culture. This is also illustrated in our results, where illness perceptions studies are included in women with breast cancer from Indonesia [18, 19] and Japan [15]. While Japanese and Dutch women with breast cancer score relatively similar on the illness perceptions measure [15], Indonesian women with breast cancer score very much lower on personal control and treatment control [18]. Cultural differences in beliefs in medical treatment and beliefs in “natural remedies, (herbs, etc.)” shape illness perceptions [Dein 35]. This topic needs much more research from an illness perceptions point of view.

Our paper fits in with comparable research in patients with other chronic somatic illnesses. For example, illness perceptions in patients with hemodialysis were shown not only to impact on QOL but also to be predicting mortality [20, 21]. Illness perceptions predict mortality in patients with cardiac valve replacement, irrespective of clinical characteristics [22]. In patients with asthma, illness perceptions were shown to be associated with various aspects of QOL [23]. The paper by Broadbent on the B-IPQ reports means and standard deviation of scores of the dimensions of the B-IPQ in patients with diabetes, asthma, colds, and myocardial infarction. Recently, van Leeuwen et al. [24] compared the B-IPQ scores of the patients with vestibular schwannoma in her study with patients with SLE, colorectal cancer, lung cancer, and melanoma. Compared with the patients in the papers by Iskandarsyah et al. [18] and Kaptein et al. [15] on women with breast cancer, the patients with breast cancer appear to score higher (better) on treatment control and personal control. Medical characteristics of the diseases under study do seem to shape illness perceptions at least in part. Cultural and psychosocial responses to various diseases in various cultures appear to be additional determinants of patterns of illness perceptions [25].

A limitation of our paper pertains to the studies included in the review. Most studies are cross-sectional and describe, therefore, associations between illness perceptions and a number of important and relevant outcome measures. It may be better to use the word concomitants rather than associations, therefore. Intervention studies are waiting to be done. For instance, Nordic Walking for women with breast cancer appears to affect participants’ perceptions about their arm/shoulder morbidity [26]. In the Nordic Walking exercises, sessions focused on upper body strength and condition. Patients’ perceptions of their arm and shoulder morbidity were assessed with the B-IPQ. Results indicated that after 10 weeks, patients’ vitality had improved, perceived shoulder symptom severity and limitations in daily activities had decreased, and range of motion of the affected shoulder improved significantly. Scores on the B-IPQ showed improvements in consequences and symptoms ([26], pp. 278–9). In an earlier study, Fischer et al. demonstrated in a longitudinal study how a psychosocial aftercare program impacted on illness perceptions and coping, and thereby on emotional well-being in women with breast cancer [3•]. The intervention program entailed nine meetings of about 2 h. Topics discussed were, for example, what is breast cancer, being diagnosed with breast cancer, coping, social support, and stress management. In addition, three types of exercise were part of the intervention: physical exercises, rational-emotive exercises, and behavioral exercises [3•, p. 529].

The research implications of our review are fairly straightforward. In a study on illness perceptions in survivors of a myocardial infarction, Broadbent et al. demonstrated in an experimental design how substituting maladaptive illness perceptions into constructive, adaptive illness perceptions resulted in an earlier return to work, earlier resumption of sexual activities, and fewer symptoms of angina [12]. In the area of breast cancer, the research group of Antoni in the USA does major work in applying cognitive behavioral therapy (CBT) in women with breast cancer [1•, 27]. Their research can be summarized as showing how applying CBT intended to change unhelpful illness perceptions resulted in more constructive illness perceptions, which in turn resulted in a better QOL and reductions in distress. In the Netherlands, the research group of Aaronson publishes similar exciting results [17]. Older patients with breast cancer frequently undergo breast amputation, while it is known that this will not influence overall survival. Therefore, other important factors such as illness perceptions should be included in the decision about the best treatment for these patients [28].

Assessing illness perceptions in order to identify patients for whom intervention in this psychosocial domain seems indicated is an important part of modern biopsychosocial care for women with breast cancer (and for any patient with a chronic somatic disease, for that matter). Additional studies are indicated in order to decide cutoff points in illness perception score where intervention seems most cost-effective and efficient.

Future research most likely will address the question of whether cognitive-behavioral interventions aimed at changing addressing unhelpful illness perceptions into more adaptive ones impact on outcome variables such as duration of recurrence free interval and even survival.

Clinically, our review and related illness perception research suggest the following:

1. Incorporate assessing illness perceptions into clinical care, similar to incorporating laboratory values into diagnostic and therapeutic policy.
2. Sensitize health care providers about the importance of illness perceptions (or explanatory models) that patients maintain.
3. Address illness perceptions that appear to hamper the uptake of adaptive behaviors.

In summary, it seems that Hippocrates is right after all: Listen to the patient’s story. Only then will you be able to help her best.

## Appendix

**Table 2:** Search strategies

Database	Search strategy	Number of references	Number of unique references
PubMed	((“illness representations”[tw] OR “illness representation”[tw] OR “disease representations”[tw] OR “disease representation”[tw] OR “illness perception”[tw] OR “illness perceptions”[tw] OR “disease perception”[tw] OR “disease perceptions”[tw] OR “illness cognition”[tw] OR “illness cognitions”[tw] OR “disease cognition”[tw]) AND (“Breast Neoplasms”[Mesh] OR “breast cancer”[tw] OR “Breast Cancers”[tw] OR “Breast Neoplasm”[tw] OR “Breast Neoplasms”[tw] OR “Breast Tumors”[tw] OR “Breast Tumor”[tw] OR “Breast Tumours”[tw] OR “Breast Tumour”[tw] OR “Mammary Neoplasm”[tw] OR “Mammary Neoplasms”[tw] OR “Mammary Carcinomas”[tw] OR “Mammary Carcinoma”[tw] OR “Cancer of Breast”[tw] OR “Mammary Cancer”[tw] OR “Tumor of Breast”[tw] OR “Breast Carcinoma”[tw] OR “Breast Carcinomas”[tw] OR “Cancer of the Breast”[tw]) AND (english[la] OR dutch[la]) AND (“2012/01/01”[PDAT] : “3000/12/31”[PDAT])) OR (((“illness representations”[ti] OR “illness representation”[ti] OR “disease representations”[ti] OR “disease representation”[ti] OR “illness perception”[ti] OR “illness perceptions”[ti] OR “disease perception”[ti] OR “disease perceptions”[ti] OR “illness cognition”[ti] OR “illness cognitions”[ti] OR “disease cognition”[ti]) AND (“Breast Neoplasms”[majr] OR “breast cancer”[ti] OR “Breast Cancers”[ti] OR “Breast Neoplasm”[ti] OR “Breast Neoplasms”[ti] OR “Breast Tumors”[ti] OR “Breast Tumor”[ti] OR “Breast Tumours“[ti] OR “Breast Tumour”[ti] OR “Mammary Neoplasm”[ti] OR “Mammary Neoplasms”[ti] OR “Mammary Carcinomas”[ti] OR “Mammary Carcinoma”[ti] OR “Cancer of Breast”[ti] OR “Mammary Cancer“[ti] OR “Tumor of Breast”[ti] OR “Breast Carcinoma”[ti] OR “Breast Carcinomas”[ti] OR “Cancer of the Breast”[ti]))	30	30
MEDLINE (OVID-version)	((“illness representations”.mp OR “illness representation”.mp OR “disease representations”.mp OR “disease representation”.mp OR “illness perception”.mp OR “illness perceptions”.mp OR “disease perception”.mp OR “disease perceptions”.mp OR “illness cognition”.mp OR “illness cognitions“.mp OR “disease cognition”.mp OR ((illness*.ti,ab OR disease*.ti,ab) ADJ4 (representat*.ti,ab OR perception*.ti,ab))) AND (exp *“Breast Neoplasms”/ OR “breast cancer”.ti,ab OR “Breast Cancers”.ti,ab OR “Breast Neoplasm”.ti,ab OR “Breast Neoplasms”.ti,ab OR “Breast Tumors”.ti,ab OR “Breast Tumor”.ti,ab OR “Breast Tumours”.ti,ab OR “Breast Tumour”.ti,ab OR “Mammary Neoplasm”.ti,ab OR “Mammary Neoplasms”.ti,ab OR “Mammary Carcinomas”.ti,ab OR “Mammary Carcinoma”.ti,ab OR “Cancer of Breast”.ti,ab OR “Mammary Cancer”.ti,ab OR “Tumor of Breast”.ti,ab OR “Breast Carcinoma”.ti,ab OR “Breast Carcinomas”.ti,ab OR “Cancer of the Breast”.ti,ab) AND (english OR dutch).la AND (2012 OR 2013 OR 2014 OR 2015).yr) OR ((“illness representations”.ti OR “illness representation”.ti OR “disease representations”.ti OR “disease representation”.ti OR “illness perception”.ti OR “illness perceptions”.ti OR “disease perception”.ti OR “disease perceptions”.ti OR “illness cognition”.ti OR “illness cognitions”.ti OR “disease cognition”.ti OR ((illness*.ti OR disease*.ti) ADJ4 (representat*.ti OR perception*.ti))) AND (exp *“Breast Neoplasms”/ OR “breast cancer”.ti OR “Breast Cancers”.ti OR “Breast Neoplasm”.ti OR “Breast Neoplasms”.ti OR “Breast Tumors”.ti OR “Breast Tumor”.ti OR “Breast Tumours”.ti OR “Breast Tumour”.ti OR “Mammary Neoplasm”.ti OR “Mammary Neoplasms”.ti OR “Mammary Carcinomas”.ti OR “Mammary Carcinoma”.ti OR “Cancer of Breast”.ti OR “Mammary Cancer”.ti OR “Tumor of Breast”.ti OR “Breast Carcinoma”.ti OR “Breast Carcinomas”.ti OR “Cancer of the Breast”.ti))	41	15
Embase (OVID version)	(((“illness representations”.mp OR “illness representation”.mp OR “disease representations”.mp OR “disease representation“.mp OR “illness perception“.mp OR “illness perceptions”.mp OR “disease perception”.mp OR “disease perceptions”.mp OR “illness cognition”.mp OR “illness cognitions”.mp OR “disease cognition”.mp OR ((illness*.ti,ab OR disease*.ti,ab) ADJ4 (representat*.ti,ab OR perception*.ti,ab))) AND (exp *“Breast Tumor”/ OR “breast cancer”.ti,ab OR “Breast Cancers”.ti,ab OR “Breast Neoplasm”.ti,ab OR “Breast Neoplasms”.ti,ab OR “Breast Tumors”.ti,ab OR “Breast Tumor”.ti,ab OR “Breast Tumours”.ti,ab OR “Breast Tumour”.ti,ab OR “Mammary Neoplasm”.ti,ab OR “Mammary Neoplasms”.ti,ab OR “Mammary Carcinomas”.ti,ab OR “Mammary Carcinoma”.ti,ab OR “Cancer of Breast”.ti,ab OR “Mammary Cancer”.ti,ab OR “Tumor of Breast”.ti,ab OR “Breast Carcinoma”.ti,ab OR “Breast Carcinomas”.ti,ab OR “Cancer of the Breast”.ti,ab) AND (english OR dutch).la AND (2012 OR 2013 OR 2014 OR 2015).yr) OR ((“illness representations”.ti OR “illness representation”.ti OR “disease representations”.ti OR “disease representation”.ti OR “illness perception”.ti OR “illness perceptions”.ti OR “disease perception”.ti OR “disease perceptions”.ti OR “illness cognition”.ti OR “illness cognitions”.ti OR “disease cognition”.ti OR ((illness*.ti OR disease*.ti) ADJ4 (representat*.ti OR perception*.ti))) AND (exp *“Breast Tumor”/ OR “breast cancer”.ti OR “Breast Cancers”.ti OR “Breast Neoplasm“.ti OR “Breast Neoplasms“.ti OR “Breast Tumors”.ti OR “Breast Tumor’.ti OR “Breast Tumours”.ti OR “Breast Tumour”.ti OR “Mammary Neoplasm”.ti OR “Mammary Neoplasms”.ti OR “Mammary Carcinomas”.ti OR “Mammary Carcinoma”.ti OR “Cancer of Breast”.ti OR “Mammary Cancer”.ti OR “Tumor of Breast”.ti OR “Breast Carcinoma”.ti OR “Breast Carcinomas”.ti OR “Cancer of the Breast”.ti))) NOT conference abstract.pt	43	6
Web of science	((TI = (“illness representations” OR “illness representation” OR “disease representations” OR “disease representation” OR “illness perception” OR “illness perceptions” OR “disease perception” OR “disease perceptions” OR “illness cognition” OR “illness cognitions” OR “disease cognition” OR ((illness* OR disease*) NEAR4 (representat* OR perception*))) AND TS = (“breast cancer” OR “Breast Cancers” OR “Breast Neoplasm” OR “Breast Neoplasms” OR “Breast Tumors” OR “Breast Tumor” OR “Breast Tumours” OR “Breast Tumour” OR “Mammary Neoplasm” OR “Mammary Neoplasms” OR “Mammary Carcinomas” OR “Mammary Carcinoma” OR “Cancer of Breast” OR “Mammary Cancer” OR “Tumor of Breast” OR “Breast Carcinoma” OR “Breast Carcinomas” OR “Cancer of the Breast”) AND la = (english OR dutch) AND py = (2012 OR 2013 OR 2014 OR 2015)) OR (TI = (‘illness representations” OR “illness representation” OR “disease representations” OR “disease representation” OR “illness perception” OR “illness perceptions” OR “disease perception” OR “disease perceptions” OR “illness cognition” OR “illness cognitions” OR “disease cognition” OR ((illness* OR disease*) NEAR4 (representat* OR perception*))) AND TI = (“breast cancer” OR “Breast Cancers” OR “Breast Neoplasm” OR “Breast Neoplasms” OR “Breast Tumors” OR “Breast Tumor” OR “Breast Tumours” OR “Breast Tumour” OR “Mammary Neoplasm” OR “Mammary Neoplasms” OR “Mammary Carcinomas” OR “Mammary Carcinoma” OR “Cancer of Breast” OR “Mammary Cancer” OR “Tumor of Breast” OR “Breast Carcinoma” OR “Breast Carcinomas” OR “Cancer of the Breast”))) OR ((TS = (“illness representations” OR “illness representation” OR “disease representations” OR “disease representation” OR “illness perception” OR “illness perceptions” OR “disease perception” OR “disease perceptions” OR “illness cognition” OR “illness cognitions” OR “disease cognition” OR ((illness* OR disease*) NEAR4 (representat* OR perception*))) AND TI = (“breast cancer” OR “Breast Cancers” OR “Breast Neoplasm” OR “Breast Neoplasms” OR “Breast Tumors” OR “Breast Tumor” OR “Breast Tumours” OR “Breast Tumour” OR “Mammary Neoplasm” OR “Mammary Neoplasms” OR “Mammary Carcinomas” OR “Mammary Carcinoma” OR “Cancer of Breast” OR “Mammary Cancer” OR “Tumor of Breast” OR “Breast Carcinoma” OR “Breast Carcinomas” OR “Cancer of the Breast”) AND la = (english OR dutch) AND py = (2012 OR 2013 OR 2014 OR 2015)) OR (TI = (“illness representations” OR “illness representation” OR “disease representations” OR “disease representation” OR “illness perception” OR “illness perceptions” OR “disease perception” OR “disease perceptions” OR “illness cognition” OR “illness cognitions” OR “disease cognition” OR ((illness* OR disease*) NEAR4 (representat* OR perception*))) AND TI = (“breast cancer” OR “Breast Cancers” OR “Breast Neoplasm” OR “Breast Neoplasms” OR “Breast Tumors” OR “Breast Tumor” OR “Breast Tumours” OR “Breast Tumour” OR “Mammary Neoplasm” OR “Mammary Neoplasms” OR “Mammary Carcinomas” OR “Mammary Carcinoma” OR “Cancer of Breast” OR “Mammary Cancer” OR “Tumor of Breast” OR “Breast Carcinoma” OR “Breast Carcinomas” OR “Cancer of the Breast”)))	33	13
COCHRANE library	(“illness representations” OR “illness representation” OR “disease representations” OR “disease representation” OR “illness perception” OR “illness perceptions” OR “disease perception” OR “disease perceptions” OR “illness cognition” OR “illness cognitions” OR “disease cognition” OR ((illness* OR disease*) NEAR4 (representat* OR perception*))) AND (“breast cancer” OR “Breast Cancers” OR “Breast Neoplasm” OR “Breast Neoplasms” OR “Breast Tumors” OR “Breast Tumor” OR “Breast Tumours” OR “Breast Tumour“ OR “Mammary Neoplasm” OR “Mammary Neoplasms” OR “Mammary Carcinomas” OR “Mammary Carcinoma” OR “Cancer of Breast” OR “Mammary Cancer” OR “Tumor of Breast” OR “Breast Carcinoma” OR “Breast Carcinomas” OR “Cancer of the Breast”)	9	5
CINAHL (EbschoHost-version)	ti or ab (“illness representations” OR “illness representation” OR “disease representations” OR “disease representation” OR “illness perception” OR “illness perceptions” OR “disease perception” OR “disease perceptions” OR “illness cognition” OR “illness cognitions” OR “disease cognition” OR ((illness* OR disease*) N4 (representat* OR perception*))) AND (“breast cancer” OR “Breast Cancers” OR “Breast Neoplasm” OR “Breast Neoplasms” OR “Breast Tumors” OR “Breast Tumor” OR “Breast Tumours” OR “Breast Tumour” OR “Mammary Neoplasm” OR “Mammary Neoplasms” OR “Mammary Carcinomas” OR “Mammary Carcinoma” OR “Cancer of Breast” OR “Mammary Cancer” OR “Tumor of Breast” OR “Breast Carcinoma” OR “Breast Carcinomas” OR “Cancer of the Breast”)	47	15
PsycINFO (EbscoHost-version)	ti or mj or ab or su (“illness representations” OR “illness representation” OR “disease representations” OR “disease representation” OR “illness perception” OR “illness perceptions” OR “disease perception” OR “disease perceptions” OR “illness cognition” OR “illness cognitions” OR “disease cognition” OR ((illness* OR disease*) N4 (representat* OR perception*))) AND (“breast cancer” OR “Breast Cancers” OR “Breast Neoplasm” OR “Breast Neoplasms” OR “Breast Tumors” OR “Breast Tumor” OR “Breast Tumours” OR “Breast Tumour“ OR “Mammary Neoplasm” OR “Mammary Neoplasms” OR “Mammary Carcinomas” OR “Mammary Carcinoma” OR “Cancer of Breast” OR “Mammary Cancer” OR “Tumor of Breast“ OR “Breast Carcinoma” OR “Breast Carcinomas” OR “Cancer of the Breast”)	30	6
Total			90
